# Pipeline Embolization Device for the Treatment of Unruptured Intracranial Dissecting Aneurysms

**DOI:** 10.3389/fneur.2021.691897

**Published:** 2021-09-14

**Authors:** Jigang Chen, Mushun Tao, Jiangli Han, Xin Feng, Fei Peng, Xin Tong, Hao Niu, Ning Ma, Aihua Liu

**Affiliations:** ^1^Beijing Neurosurgical Institute, Capital Medical University, Beijing, China; ^2^Department of Interventional Neuroradiology, Beijing Tiantan Hospital, Capital Medical University, Beijing, China; ^3^Department of Neurosurgery, First Hospital of Shanxi Medical University, Taiyuan, China; ^4^Department of Neurosurgery, The Third Xiangya Hospital, Central South University, Changsha, China; ^5^Department of Neurosurgery Beijing Hospital, National Center of Gerontology, Beijing, China

**Keywords:** unruptured intracranial dissecting aneurysms, pipeline endovascular device, outcomes, complications, treatment

## Abstract

**Background:** Intracranial dissecting aneurysms (IDAs) are rare but pose significant challenges to treatment. The pipeline embolization device (PED) has been demonstrated to be an effective treatment option with excellent outcomes. Herein, we report our experience with patients treated with the PED for unruptured IDAs.

**Methods:** We retrospectively reviewed our hospital database and identified patients who were treated with PEDs for unruptured IDAs between March 2016 and September 2020. Data including demographics, clinical presentation, aneurysm characteristics, procedural details, intra- or peri-procedural complications, and follow-up details were collected.

**Results:** Eighty patients (61 men, 76.25%) were treated with PED for unruptured IDAs. The most common symptoms were headache (34, 42.5%), dizziness (29, 36.25%), and nausea or vomiting (15, 18.75%). Of these patients, 73 had one aneurysm, and seven harbored two aneurysms. All of them achieved successful PED deployment. Six patients experienced intra- or peri-procedural complications including perforator artery occlusion, thromboembolic, hemorrhagic events, and falling of the stent into the aneurysm sac. Follow-up with digital subtractive angiography was available for 29 patients with a median of 6 months, and 28 (96.56%) patients had aneurysm occlusion. Late thrombosis occurred in four patients, and two of them had unfavorable outcomes. Clinical follow-up showed that a favorable clinical outcome was achieved in 76 (95%) patients, and the mortality rate was 3.75%.

**Conclusion:** Treating unruptured IDAs is safe and effective with long-term favorable clinical and angiographic outcomes. However, the complications of this treatment should be noted. Careful selection of appropriate patients and individualized antiplatelet therapy might be needed.

## Introduction

Intracranial dissecting aneurysms (IDAs) are uncommon types of cerebrovascular lesions caused by a disruption of the internal elastic lamina and account for only 3% of all intracranial aneurysms (1). Even though they are less frequent than saccular aneurysms, IDAs have been recognized as an important source of subarachnoid hemorrhage (SAH) in children, young adults, and middle-aged adults, especially in the East Asian population (2, 3). The risk of SAH has shifted treatment for unruptured IDAs away from initial conservative therapy toward more invasive approaches. However, these lesions still pose a major challenge to endovascular treatment due to their unique location and anatomic characteristics (4, 5).

The flow-diverting pipeline embolization device (PED) has been approved in 2011 by the United States Food and Drug Administration for the treatment of large or giant wide-necked intracranial aneurysms of the internal carotid artery. Later, multiple studies demonstrated the safety and efficacy of expanding indications for PED including ruptured aneurysms, blister aneurysms, and dissecting aneurysms (6). The PED can be used as an endoluminal reconstruction device that preserves the parent artery and major side branches. These characteristics of a PED make it ideal for the treatment of IDAs.

The treatment of unruptured IDAs with a PED has been reported by several studies (7–14). The outcomes seemed to be excellent, and no unfavorable outcomes or adverse events were reported by these studies (6). However, most of these studies have not investigated the use of a PED specifically for unruptured IDAs (8, 9, 12, 13), and all of them used a small number of cases. Owing to these limitations, we present a retrospective analysis of the procedure-related complications, angiographic outcomes, and clinical outcomes for patients with unruptured IDAs who received PED treatment in a high-volume center.

## Methods

### Study Design

This study was approved by the institutional review board of Beijing Tiantan Hospital, and the need for informed consent was waived due to the retrospective design. We reviewed our hospital database to identify consecutive patients who were admitted to our department for the treatment of unruptured IDAs between March 2016 and September 2020.

The inclusion criteria for this study were as follows: (1) IDAs diagnosed by digital subtraction angiography (DSA). The diagnosis for IDAs was established according to imaging findings including intimal flap, pearl and string sign, double-lumen sign, luminal dilation adjacent to a stenotic segment, or a simple fusiform dilation with delayed clearance of contrast media (1); (2) aneurysms treated by PED alone or a PED with adjunctive coiling. The exclusion criteria were as follows: (1) aneurysms treated by non-PED approaches; (2) history of SAH; (3) major diseases such as stroke, cerebral artery stenosis, injury, or tumor that would affect patients' outcomes; and (4) incomplete clinical data. Clinical and angiographic data were reviewed by two experienced neurologists. Finally, 80 consecutive patients were included in this study.

The following variables were collected for eligible patients: demographics, aneurysm characteristics, antiplatelet treatment, procedural details, immediate or delayed complications, radiographic data, and functional outcomes. Complications were considered if new symptoms emerged that could attribute to either thromboembolism or hemorrhage.

### Treatment Details

Before treatment, each case was discussed with neurovascular team members, and the treatment decision was made based on demographics, symptoms, location, and morphology. There were no strict criteria for aneurysm size.

Patients in this series were preloaded with a daily oral antiplatelet regimen consisting of 100 mg of aspirin and 75 mg of clopidogrel for 5 days before treatment. Patients' reactivity to these two antiplatelet drugs was routinely tested; if a low response to clopidogrel was indicated, it was replaced with ticagrelor.

Treatments were performed with the patient under general anesthesia and via a transfemoral approach, and systemic heparinization was administered after placement of the sheath. The Marksman microcatheter (Medtronic, Dublin, Ireland) was manipulated under high-magnification fluoroscopic roadmap control across the aneurysm neck. We attempted to cover the aneurysm neck with a minimal number of PEDs, and multiple PEDs were considered when a single PED could not bridge the wide neck. The PED position was adjusted from multiple angles before releasing it carefully. Stent apposition was evaluated by DynaCT (Siemens Healthcare GmbH, Erlangen, Germany). Flow stagnation inside the aneurysm was assessed to decide whether to insert additional coils. After the operation, patients were asked to take 75 mg of clopidogrel and 100 mg of aspirin daily for 6 months for the rest of their life.

### Assessment and Follow-Up

Technical success was defined as complete coverage of the aneurysm neck after PED deployment and preserved patency of the parent artery without clinically evident adverse events. Patients were advised to undergo both clinical and angiographic follow-up 3, 6, and 12 months after the treatment and annually thereafter. An independent neurologist was responsible for the neurologic assessment. Any residual filling of the aneurysms was interpreted as incomplete occlusion. Functional outcomes were evaluated using the modified Rankin scale (mRS), of which a score of 0 to 2 was defined as a favorable outcome and a score of 3–6 as an unfavorable outcome. This score was obtained during a follow-up visit at our clinic or by telephone interview for those referred from distant locales.

## Results

Over a 5 year study period, 80 patients (61 male, 76.25%) were treated with PEDs due to unruptured IDAs (Table 1). Their age ranged from 10 to 71 years (median, 53 years), and 61 (76.25%) of them were male. The most common symptoms were headache (34, 42.5%), dizziness (29, 36.25%), and nausea or vomiting (15, 18.75%).

**Table 1:** Baseline characteristics.

**Number of patients**	***N* = 80**
**Sex**	
Male	61 (76.25%)
Female	19 (23.75%)
Age (years), median (IQR)	53 (47–56)
**Symptoms**	
Headache	34 (42.5%)
Dizziness	29 (36.25%)
Nausea or vomiting	15 (18.75%)
**Pretreatment mRS**	
0	31 (38.75%)
1	44 (55%)
2	5 (6.25%)
Hypertension	44 (55%)
Diabetes mellitus	7 (8.75%)
Hyperlipidemia	7 (8.75)
Smoking	28 (35%)
Alcohol	20 (25%)
Number of patients with a single aneurysm	73 (91.25%)
Number of patients with two aneurysms	7 (8.75%)
**Aneurysm size**	
Small (<10 mm)	30 (34.48%)
Large (10–25 mm)	50 (57.47%)
Giant (>25 mm)	7 (8.04%)
**Aneurysm location**	
Vertebral artery	75 (86.21%)
Basilar artery	4 (4.6%)
Vertebrobasilar junction	3 (3.45%)
Middle cerebral artery	3 (3.45%)
Posterior cerebral artery	1 (1.15%)
Carotid artery	1 (1.15%)
**Procedure details for patients with a single aneurysm**	
PED alone	61
PED with adjunctive coiling	12
**Procedures details for patients with two aneurysms**	
Both aneurysms treated	4
One aneurysm treated	3
Ischemic complications	4 (5%)
Hemorrhagic complications	1 (1.25%)
Number of patients with radiological follow up	29 (36.25%)
**Length of radiological follow-up (months), median (IQR)**	
**Complete occlusion of aneurysms**	
Length of clinical follow-up (months), median (IQR)	26 (16–37)
**Follow-up mRS**	
0	56 (70%)
1	18 (22.5%)
2	2 (2.5%)
5	1 (1.25%)
6	3 (3.75%)

*IQR, interquartile range; PED, pipeline embolization device; mRS, modified Rankin Scale*.

Most of those patients had IDAs that compromised the intradural segment of the vertebral artery (68, 85%), basilar artery (4, 5%), vertebrobasilar junction (3, 3.75%), middle cerebral artery (3, 3.75%), posterior cerebral artery (1, 1.25%), and carotid artery (1, 1.25%). Among these patients, seven had multiple aneurysms: one patient had two aneurysms on one vertebral artery, while six patients had an aneurysm on both vertebral arteries. Therefore, 80 patients harbored a total of 87 aneurysms. Fifty (57.47%, 50/87) aneurysms were classified as large (10–25 mm), 30 (34.48%, 30/87) as small (<10 mm), and seven (8.04%, 7/87) as giant (>25 mm).

None of the patients in our cohort were treated previously with other stents or coils. Of the 73 patients with a single aneurysm, 61 had PED placement alone and 12 had adjunctive coiling. A total of 80 devices were used to treat 73 IDAs. Four patients with multiple aneurysms had both aneurysms treated, and two of them had staged treatment. The other three patients with multiple aneurysms had only one aneurysm treated. Successful deployment of the PED was achieved for all patients, and only two of them required a second attempt. Two patients presenting with an aneurysm at the vertebrobasilar junction had PED deployment on one side and vertebral artery sacrifice on the other.

Six patients experienced intra- or peri-procedural complications, and one had perforator artery occlusion during the procedure. Tirofiban was administered after the exclusion of hemorrhage confirmed by DynaCT; this patient did not develop any new symptoms after treatment and recovered well. One encountered in-stent thrombosis during the procedure, and tirofiban was administered proximal to thrombus intra-arterially through a microcatheter; the patient recovered well at follow-up. Two patients suffered from thromboembolic complications within 2 days after the operation. One of these two patients underwent vertebral artery sacrifice, and they recovered well at discharge. However, that patient died due to in-stent thrombosis 10 months later. The other patient who experienced a thromboembolic event managed to have a full recovery at follow-up. One patient presented with intracranial hemorrhage 1 day after the operation and died at discharge. One patient had the stent fall into the aneurysm sac 4 days after the procedure, and basilar artery sacrifice was performed. The patient recovered well at a long-term follow-up with an mRS score of 2.

At least one DSA follow-up was available among 29 patients (37.18%, 29/78). The median follow-up duration was 6 months (range, 3–18 months). We selected the final DSA follow-up of every patient as the timepoint at which to evaluate the efficacy of PED. Only one patient had obvious aneurysm residual on follow-up, and the occlusion rate was 96.56%. Two patients were confirmed to have vertebral artery occlusion on the side of PED placement, and they presented with mild-to-moderate dizziness.

The median clinical follow-up was 26 months (range, 3–61 months) for patients who were alive at discharge. A favorable clinical outcome was achieved in 76 (95%) of patients. One patient with basilar artery IDAs had cerebellar infarction diagnosed at the local hospital 5 months after the treatment and was severely disabled (mRS 5). One patient died suddenly at the last follow-up due to unknown reasons 1 month after discharge. The overall unfavorable rate was 5%, and the mortality rate was 3.75%.

### Illustrative Case 1

**Figure 1:**
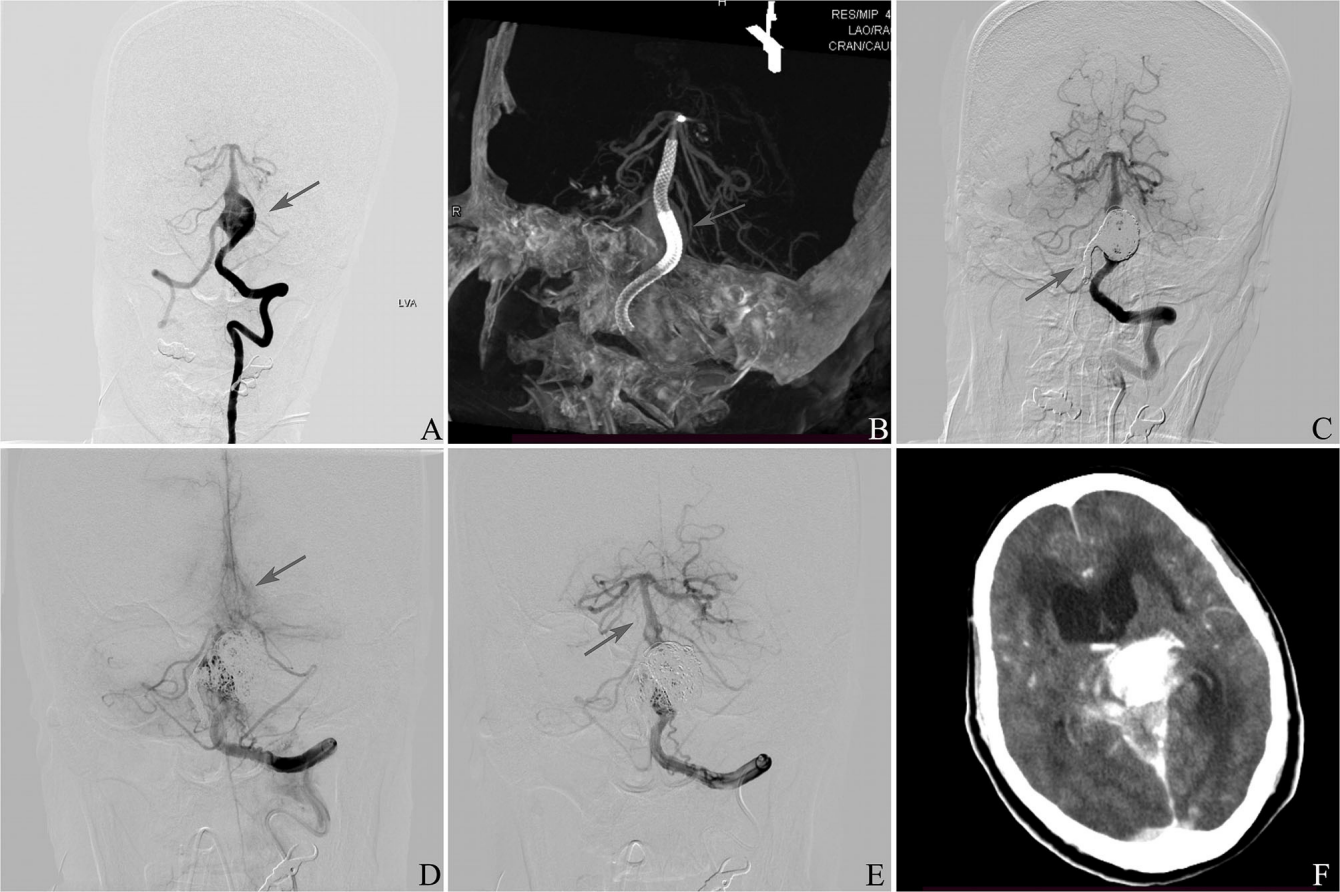
**(A)** Frontal vertebral arteriograms showing a dissecting aneurysm at the vertebrobasilar junction (arrow). **(B)** Placement of pipeline embolization device (arrow). **(C)** Sacrifice of right vertebral artery (arrow). **(D)** Occlusion of basilar artery 10 months later after discharge (arrow). **(E)** Reopening of the basilar artery (arrow). **(F)** Computed tomography showing the brain stem, thalamus, and subarachnoid hemorrhage.

This patient was a 61 year-old female in whom an IDA was accidentally discovered (Figure 1). On the initial diagnostic angiogram, the presence of an IDA at the vertebrobasilar junction was confirmed. She had a normal response to aspirin and clopidogrel. A PED was placed, and the right vertebral cerebral artery was sacrificed. She experienced a thromboembolic event 1 day after the procedure and recovered without any complications at discharge. The DSA follow-up 6 months later showed that the aneurysm was occluded completely. This patient had a sudden loss of consciousness 10 months later. DSA confirmed the occlusion of the basilar artery due to in-stent thrombosis, and intra-arterial thrombolysis was administered immediately. The basilar artery was reopened completely after the procedure, and computed tomography showed the brain stem, thalamus, and SAH. She died 1 day later.

### Illustrative Case 2

**Figure 2:**
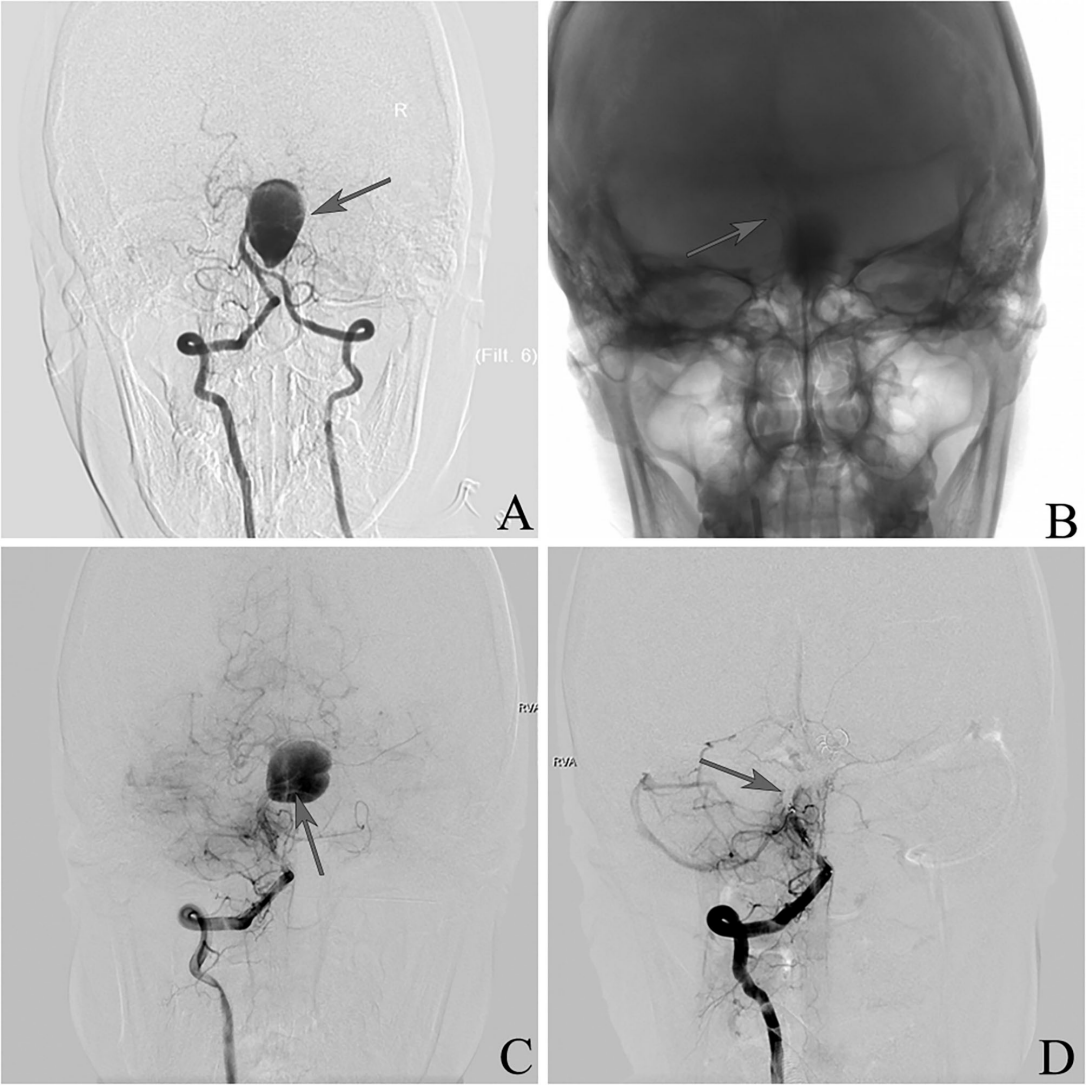
**(A)** Frontal vertebral arteriograms showing a dissecting aneurysm at the upper basilar trunk (arrow). **(B)** Placement of pipeline embolization device (arrow). **(C)** Pipeline embolization device falling into aneurysm sac (arrow). **(D)** Sacrifice of the basilar artery (arrow).

This was a 37-year-old female presenting with a right occipital headache (Figure 2). Angiography demonstrated a giant IDA involving the upper basilar trunk. Two PEDs were deployed in the right position. She developed a severe progressive headache and vomiting the next 4 days after the procedure. DSA showed the PEDs had fallen into the aneurysm sac, and basilar artery sacrifice was then performed. The patient had an uneventful recovery with mild disability (mRS 2) at discharge, and she remained the same at follow-up.

## Discussion

Currently, the natural history of unruptured IDAs is still unclear, and their management remains a dilemma (15). Conservative treatment with anticoagulation therapy was initially adopted and resulted in a benign clinical outcome, suggesting that intervention is not always required and that close follow-up is reasonable (16, 17). However, the risk of bleeding from unruptured IDAs was proved to be higher than previously thought and has been an important source of SAH (5, 18). Symptoms due to mass effects or aneurysms with large size or persistent dilation further supported the argument for definitive treatment of unruptured IDAs.

Treatment of IDAs is regarded as a technical challenge due to their histopathological features and localization. There are several treatment strategies for IDAs including proximal occlusion, trapping with or without bypass, clipping or wrapping of the aneurysm sac, stent-assisted coil embolization, and stent monotherapy with flow diverters. Since its origin, flow diverters, especially the PED, have emerged as an attractive therapeutic option for these challenging lesions. The PED consists of a tightly braided alloy and has low porosity. It diverts blood flow and allows blood to cross the interstices. The metal surface area acts as a scaffold for intraluminal reconstruction. Therefore, the PED can facilitate aneurysm exclusion and also preserve important functional perforators (19).

The initial experience of using PED for the treatment of unruptured IDAs was shared by de Barros Faria et al. (13). Eleven patients with unruptured IDAs were treated in that study. Though the complications after treatment were not reported specifically, all patients achieved a good clinical outcome at a short-term follow-up. Later, Yeung et al. reported the long-term outcome of four patients with unruptured IDAs of the vertebral artery receiving endovascular reconstruction using PEDs (14). No periprocedural complication was encountered, and no patient showed any recurrence, in-stent thrombosis, or side-branch occlusion at angiographic follow-up at a mean of 22 months after treatment. All of them had favorable outcomes with an mRS score of 0 at long-term clinical follow-up. Fischer et al. reported the largest number of cases using PEDs for the treatment of intra- and extracranial fusiform and dissecting aneurysms (8). In this case series, 69 aneurysms were treated, and 31 were classified as dissecting. The morbidity and mortality rates in this entire series were 5 and 8%, respectively. However, the outcomes for patients with unruptured IDAs were not reported. A few other studies with a small number of cases also investigated the efficacy and safety of the PED for the treatment of unruptured IDAs, and the results seem excellent (6, 7, 9–12).

According to our knowledge, we reported the largest number of cases, and 80 consecutive patients harboring 87 unruptured IDAs were included in this study. A total of 84 aneurysms were treated by a PED or a PED with an adjunctive coil. Technical success was achieved for all these patients. Twenty-nine patients had DSA follow-up, and 28 (96.56%) of them had aneurysm occlusion. A favorable clinical outcome was achieved in 76 (93.75%) of patients with a median follow-up of 26 months, and the mortality rate was 3.75%. Overall, the results of our cohort proved that using PEDs for the treatment of unruptured IDAs is effective and safe.

However, special attention should be paid to the complications of PED treatment for unruptured IDAs even though they were not reported previously. In-stent thrombosis, thromboembolic events, and hemorrhagic events are feared complications. In our cohort, six patients experienced intra- or peri-procedural complications. Intraprocedural events occurred in two patients. One had a perforator artery occlusion, and another had in-stent thrombosis. After the procedure, two patients suffered from thromboembolic events, and one from a severe hemorrhagic event. One had the stent fall into the aneurysm sac, and the basilar artery was ultimately sacrificed. One of these six patients died due to hemorrhage at discharge. These events prove that careful selection of patients appropriate for PED treatment and intensive care after the procedure are needed.

Late thrombosis is another complication that should be noted even though it is considered a rare event (20). In our study, two patients developed vertebral artery occlusion on the side of PED placement, and they had mild-to-moderate dizziness. Moreover, at the follow-up, one patient died due to basilar artery occlusion caused by in-stent thrombosis 10 months later, and one patient had a severe disability due to cerebellar infarction 5 months later after the PED placement at the basilar artery. All these patients were compliant with antiplatelet treatment. Therefore, late thrombosis after PED placement, especially in the posterior circulation, might not be rare as previously thought. Special attention should be paid to antiplatelet therapy, and it might need to be individualized.

This study has several major limitations. First, the DSA imaging follow-up was only available for 29 patients, making it difficult to evaluate the effectiveness of aneurysm occlusion after PED placement. Second, this was a single-center study, limited by its retrospective nature and by the inherent bias of this kind of study design.

## Conclusion

Reconstruction using a PED is safe and effective in the treatment of unruptured IDAs, showing favorable long-term clinical and angiographic outcomes. However, the complications of this treatment should be noted. Careful selection of appropriate patients and individualized antiplatelet therapy might be needed.

## Data Availability Statement

The raw data supporting the conclusions of this article will be made available by the authors, without undue reservation.

## Ethics Statement

The studies involving human participants were reviewed and approved by Beijing Tiantan Hospital. Written informed consent to participate in this study was provided by the participants' legal guardian/next of kin. Written informed consent was obtained from the individual(s) for the publication of any potentially identifiable images or data included in this article.

## Author Contributions

JC conducted the study design and prepared the manuscript. MT and JH collected the data and performed the clinical follow-up. XF, FP, XT, HN, and NM reviewed the data. AL reviewed and finalized the manuscript. All authors contributed to the article and approved the submitted version.

## Funding

Funding was provided by the Beijing Science and Technology Planning Project (No. Z181100009618035).

## Conflict of Interest

The authors declare that the research was conducted in the absence of any commercial or financial relationships that could be construed as a potential conflict of interest.

## Publisher's Note

All claims expressed in this article are solely those of the authors and do not necessarily represent those of their affiliated organizations, or those of the publisher, the editors and the reviewers. Any product that may be evaluated in this article, or claim that may be made by its manufacturer, is not guaranteed or endorsed by the publisher.
