# Corrigendum: Pipeline Embolization Device for the Treatment of Unruptured Intracranial Dissecting Aneurysms

**DOI:** 10.3389/fneur.2021.824841

**Published:** 2022-01-05

**Authors:** Jigang Chen, Mushun Tao, Jiangli Han, Xin Feng, Fei Peng, Xin Tong, Hao Niu, Ning Ma, Aihua Liu

**Affiliations:** ^1^Beijing Neurosurgical Institute, Capital Medical University, Beijing, China; ^2^Department of Interventional Neuroradiology, Beijing Tiantan Hospital, Capital Medical University, Beijing, China; ^3^Department of Neurosurgery, First Hospital of Shanxi Medical University, Taiyuan, China; ^4^Department of Neurosurgery, The Third Xiangya Hospital, Central South University, Changsha, China; ^5^Department of Neurosurgery Beijing Hospital, National Center of Gerontology, Beijing, China

**Keywords:** unruptured intracranial dissecting aneurysms, pipeline endovascular device, outcomes, complications, treatment

In the published article, there was an error in affiliation 3, as published. Instead of “Department of Neurosurgery, Third Hospital of Shanxi Medical University, Taiyuan, China,” it should be “Department of Neurosurgery, First Hospital of Shanxi Medical University, Taiyuan, China.”

The authors apologize for this error and state that this does not change the scientific conclusions of the article in any way. The original article has been updated.

## Publisher's Note

All claims expressed in this article are solely those of the authors and do not necessarily represent those of their affiliated organizations, or those of the publisher, the editors and the reviewers. Any product that may be evaluated in this article, or claim that may be made by its manufacturer, is not guaranteed or endorsed by the publisher.

